# Decrease in Self-Reported Tanning Frequency among Utah Teens following the Passage of Utah Senate Bill 41: An Analysis of the Effects of Youth-Access Restriction Laws on Tanning Behaviors

**DOI:** 10.1155/2014/839601

**Published:** 2014-08-20

**Authors:** Rebecca G. Simmons, Kristi Smith, Meghan Balough, Michael Friedrichs

**Affiliations:** Utah Department of Health, Salt Lake City, UT 84116, USA

## Abstract

*Introduction*. Adolescent use of indoor tanning facilities is associated with an increased risk in later development of melanoma skin cancers. States that have imposed age restrictions on access to indoor tanning generally show lower self-reported rates of indoor tanning than states with no restrictions, but currently no studies have assessed indoor tanning use before and after such restrictions. *Methods*. In 2013, we compared self-reported indoor tanning data collected in the Prevention Needs Assessment (PNA) survey in 2011 to PNA 2013 data. We also assessed predictors of continued tanning after passage of the bill. *Results*. Prior to the passage of Senate Bill 41, 12% of students reported at least one incident of indoor tanning in the past 12 months. After passage, only 7% of students reported indoor tanning in the past 12 months (*P* < 0.0001). Students who continued indoor tanning were more likely to be older and female and to engage in other risk behaviors, including smoking and alcohol use. Lower parental education levels were also associated with continued tanning. *Conclusion*. Indoor tanning restrictions showed beneficial impact on tanning rates in adolescents in Utah. Stricter restrictions may show even greater impact than restrictions that allow for parental waivers. Stronger enforcement of bans is needed to further reduce youth access.

## 1. Introduction

Unlike many cancers which have seen a decrease in incidence and mortality over the past thirty years, incidence of melanoma skin cancer has been steadily increasing in the United States [1]. Incidence rates of melanoma skin cancer in Utah are 61% higher than the national average, with an incidence rate of 31 per 100,000 people, compared to the national rate of 19.3 per 100,000 people between 2006 and 2010 [2, 3]. Utah's melanoma mortality is also 30% higher than the national average (3.5 per 100,000 Utahns compared to 2.7 per 100,000 people nationally) [2, 3].

Melanoma is typically diagnosed later in life, but risks to adolescents and young adults have been increasing and melanoma is currently the third most common cause of cancer in individuals aged 15 to 39 years old [4]. Unlike most types of adolescent cancer, which are largely caused by genetic susceptibility, melanoma is associated with both genetic predisposition and behavioral risk factors, including the use of indoor tanning facilities [4, 5]. Indoor tanning before age 35 has been shown to increase the risk of melanoma and other skin cancers, including squamous and basal cell carcinomas [5–7], yet interventions aimed at reducing indoor tanning have shown mixed results [8–10]. Increasingly, legislative action banning teen access to indoor tanning salons has been seen as a direct way to reduce future cancer [11].

Currently, 34 states have laws in place that restrict teen access to tanning facilities in one way or another [12, 13]. Most of these laws contain exceptions that allow indoor tanning for teens who obtain physician notes or parental waivers, although, by the end of 2014, eight states (California, Illinois, Louisiana, Minnesota, Nevada, Oregon, Texas, and Vermont) will have instituted bans on* any* access to tanning facilities for those under 18. In 2014, a study by Guy et al. suggested that states with indoor tanning restrictions had significantly lower self-reported tanning rates than states without such restrictions [14]. Overall, the study found a 30% decrease in teen self-reported indoor tanning in states with any tanning laws, and a 42% decrease in self-reported indoor tanning in states with systems access, parental permissions, and age restriction laws [14]. To date, however, no study has shown the impact to adolescent indoor tanning rates before and after the passage of legislative restrictions.

The current study assesses changes in self-reported indoor teen tanning behaviors before and after the passage of Utah Senate Bill 41 in 2012, which stipulates that individuals under the age of 18 are forbidden from using indoor tanning facilities unless (1) they obtain a note from a physician or (2) they are accompanied at each tanning visit by a parent or guardian who signs a waiver on their behalf [15]. To our knowledge, this study represents the first study assessing change in teen self-reported indoor tanning behaviors immediately before and after the passage of indoor tanning restriction legislation in the United States.

## 2. Methods

Our study compares self-reported indoor tanning prevalence among teens in 2011 (prior to legislation) to prevalence in 2013 (after legislation), using Prevention Needs Assessment (PNA) survey data. Due to the deidentified, public nature of our data, no IRB approval was necessary for our study.

### 2.1. Survey Instrument

The Prevention Needs Assessment (PNA) survey is a biannual cross-sectional behavioral risk survey that is conducted as part of the Student Health and Risk Prevention (SHARP) statewide survey. The PNA survey collects self-reported data from adolescents in grades 6, 8, 10, and 12 on issues such as mental health, suicidality, health and fitness, family life, academic attitudes, drug use, and other behavioral issues. The survey is anonymous and students are informed that the answers they provide will not be traceable back to them. In order to participate in the survey, students had to return signed, parental consent forms. The survey is stratified by school district and weighted to adjust for differential response rates by grade, sex, and school district.

Both the 2011 and the 2013 PNA included the question, “during the past 12 months, how many times did you use an indoor tanning device such as sunlamps, sunbed, or tanning booth (do not include spray tan)?” Answer options included “0 times, 1 or 2 times, 3 to 9 times, 10 to 19 times, 20 to 39 times, and 40 or more times.”

### 2.2. Participants

Participants were all Utah teens in participating school districts and charter schools who were eligible for either the 2011 survey or the 2013 survey. Inclusion criteria for the survey were those adolescents in the appropriate grades, with parental permission, who were in attendance on the survey day. Exclusion criteria were those individuals who did not obtain parental permission to participate, those not in the surveyed grade levels, who were absent during the survey day, or who personally declined participation. At the end of the survey, teens were asked to report whether or not they had been honest in their survey answers. For our data, teens who reported not being honest or whose data showed high probability of dishonesty (e.g., reporting “Yes” on all questions) were excluded from the analysis. After excluding dishonest students, survey response rates were 65.1% and 69.8% in 2011 and 2013, respectively.

### 2.3. Statistical Analyses

In order to determine whether self-reported indoor tanning frequency had decreased between the 2011 PNA survey and the 2013 survey, we conducted Wald *χ* ^2^ tests comparing self-reported tanning frequencies by demographic factors from 2011 to 2013. We also conducted Wald *χ* ^2^ tests to assess whether there were demographic changes to the survey population between 2011 and 2013.

We were interested in examining factors in the 2013 PNA survey associated with continued indoor tanning use after the 2012 legislation. Prior to performing any analyses, we identified variables that were of interest or had previously been showhn to be associated with higher indoor tanning, including age, sex, race, parental education level, risk-seeking behaviors including alcohol and drug use, self-esteem measures, and anxiety measures [4]. Univariate logistic regression analyses were conducted to identify those variables showing significance (*P* ≤ .05). Variables meeting significance in the univariate analyses were included into the multivariate model using backward stepwise logistic regression.

All analyses were performed using survey procedures in SAS 9.3.

## 3. Results

Overall, self-reported indoor tanning frequency significantly decreased in both sexes and across grades between 2011 and 2013 (Table 1). In 2011, 12.0% of students reported indoor tanning at least once in the past 12 months, compared to 7.7% in 2013 (*P* < 0.0001). No significant population differences were found between the years with regard to sex (*P* = 0.820), grade (*P* = 0.971), race (*P* = 0.637), or parental education (*P* = 0.394). Significant decreases (*P* ≤ 0.05) in indoor tanning after legislation appeared with relative consistency across demographic groups, with nonsignificant decreases most likely being due to smaller sample sizes for some subpopulations. The exception to this was when stratifying by local health district, wherein sample size did not appear to be a factor.

Table 2 provides odds ratios (OR's) and 95% confidence intervals (CI's) for the full multivariate logistic regression analysis of predictors of continued tanning in 2013. Many predictors of postlegislation indoor tanning use, including sex, grade level, parental education level, race/ethnicity, substance use, and health district remained significant in the full model. Obesity did not remain significant in the full model but emerged as a significant predictor in an all-girls model, with individuals who were obese being less likely to report indoor tanning than those who were not obese (OR: 0.68, 95% CI: 0.47, 0.97).

## 4. Discussion

In 2011, approximately 12% of all Utah teens reported at least some form of indoor tanning within the past year. Following the passage of the legislative tanning restrictions in 2012, only 7.7% of teens reported indoor tanning within the past year. Guy et al. reported a national decrease of 42% in states with stricter restriction policies [14]; Utah's law, which allows some leniency with parental consent/waivers, represents a middle ground in restriction policy and shows a smaller effect (36% decrease) than perhaps states such as California, which have implemented complete bans for individuals under 18. To our knowledge, this is the first study to demonstrate a significant decrease in risk behavior following the passage of indoor tanning restrictions. Our study also demonstrates a type of policy-related dose-response relationship, consistent with prior research, suggesting that while moderate restrictions do decrease indoor tanning, stricter bans may have larger effect. Such information has important implications for health policy makers, legislators, and other individuals interested in promoting sun safety behaviors in youth.

Policy restrictions on teen indoor tanning have been compared to legal restrictions placed on teen access to tobacco, in that both are legally enforced limitations on youth to known carcinogens. Yet, while both teen smoking and indoor tanning use have been the subject of legislative policy, resulting restrictions have primary differences in how they are enforced. Policy enforcement procedures, such as compliance checks and active enforcement of tobacco sales, have resulted in a sustained decrease in teen tobacco consumption [16, 17]. Currently, no ban enforcement policies exist in Utah to assure that tanning facilities comply with age-restrictions. A 2007 study conducted by the University of Utah on tanning facility compliance to prior (and less restrictive) age-bans found that only 27% of facilities in Salt Lake County were compliant with parental consent regulations [18]. Similar national studies have found low levels of regulation compliance from tanning facilities in states with some form of teen access bans [19–21]. Enforcement of current indoor tanning bans represents an important component of policy success, nationwide.

Although overall fewer students reported indoor tanning after the legislation, there was variation in the decrease by local health district, which also remained a significant predictor of self-reported indoor tanning after legislation. It is noteworthy that local health districts that did not see a significant decrease in indoor tanning are also districts that tend to be rural. The population of Utah is largely concentrated along the mountain range of the Wasatch Front, with the majority of the total population residing within a few health districts (including Weber-Morgan, Davis, Salt Lake, and Utah County). Continued indoor tanning in more remote areas may be indicative of less oversight or enforcement in these areas or less access to media/education informing about the legislation.

Characteristics of teens who reported indoor tanning in 2013, despite legislative restrictions, mirrored outcomes of previous research. The likelihood of using indoor tanning facilities was higher in teen girls than in teen boys and increased with age, with high school seniors being the most likely to report tanning after the legislation [4, 11, 22, 23]. Teens with parents who were college graduates were less likely to continue indoor tanning in 2013 than those whose parents had not graduated from college, suggesting that parental influence plays a role in indoor tanning behavior. Previous studies have also found the correlation between parental education levels and tanning behaviors, suggesting that parental education about the dangers of indoor tanning use is an important public health component of promoting youth sun safety behavior [11, 23]. Unlike other states, such as California, that restrict any access to indoor tanning facilities in those under 18, the Utah law allows parents to sign a waiver at a tanning facility that allows temporary access to indoor tanning. Future research comparing legislative bans that do not provide parental exceptions to those that do could provide insight into the effect that such exceptions have in furthering risk behaviors.

Teens who reported engaging in risky behaviors like smoking and alcohol use were more likely to report indoor tanning than those who did not engage in other risk behaviors, though smoking fell out in the full regression model (most likely due to interaction with alcohol use). This association has been previously identified in other studies; risk taking behaviors may have some association with self-image [24]. In previous research teens have reported tanning in order to address body image issues, including a desire to look thinner and feel more confident about their bodies [23, 24]. Body image issues, including weight control, are also a primary factor in teenage decisions to smoke [25, 26]. Though underlying issues at play with such risk behaviors may not fully be understood, the association between indoor tanning use and tobacco and alcohol use could present opportunities for partnerships between cancer prevention and tobacco/alcohol cessation partners in interventions aimed at teen risk behavior reduction.

The current study had several limitations. Due to the cross-sectional nature of the PNA survey, it is impossible to say with surety that the decrease in self-reported indoor tanning use in teens was solely the result of the legislative restrictions. Sun safety education campaigns have been an ongoing part of state cancer prevention efforts and it is possible that education on the dangers of indoor tanning or other sun safety activities contributed to the reduction.

Additionally, the legislation did result in a considerable amount of news coverage over the course of the bill's proposal and passage, including at least seven television news stories on prominent news channels and more than a dozen in-depth articles in local newspapers and online. As such, it is possible that teens reporting indoor tanning use in 2013 were aware of the ban and reported tanning less as a result of awareness about the restrictions rather than an actual decrease in use. However, the PNA survey asks many questions that could be considered sensitive, including questions about things like drug use, dating violence, and self-harm behaviors. The survey goes through a rigorous evaluation process to identify students who may not have answered honestly (e.g., answering “no” to questions about alcohol use and then reporting drinking in other questions). Additionally, the PNA survey asks students whether or not they have answered questions honestly, excluding surveys from students who report dishonesty. Given the protocols to identify students who were being dishonest and the anonymity of the survey, it is likely that most students were not dishonest in their report.

To our knowledge, this study provides the first look at the effect of a state-specific legislative restriction to teen indoor tanning in the United States. Although limitations exist, this study does provide evidence to suggest that bans on indoor tanning access do appear to affect teen tanning behaviors within a short period of time. Such findings represent unique feedback for health policy makers, legislators, and others who make decisions regarding cancer prevention. While bans may decrease teen indoor tanning use, more action is needed to support such efforts, including ban enforcement measures and behavioral risk interventions that comprehensively target related risk behaviors, like tanning and alcohol use.

## Figures and Tables

**Table 1:** Comparisons in self-reported indoor tanning between PNA surveys 2011–2013 before and after passage of SB 41.

	2011 total^a^	2011 indoor tanned	2013 total^a^	2013 indoor tanned	Chi-square	*P* value
Sex						
Female	8,316	1420 (17.6%)	8004	882 (11.7%)	10.2	0.002
Male	7,293	442 (6.4%)	7180	276 (3.8%)	9.64	0.002
Combined	15,609	1862 (12%)	15,184	1158 (7.7%)	11.37	<0.0001
Grade Level						
8th grade	6,035	332 (5.2%)	6,186	228 (3.3%)	7.8	0.005
10th grade	5,389	669 (11.9%)	5,069	417 (7.4%)	8.9	0.003
12th grade	4,187	861 (19.6%)	3,970	516 (12.6%)	9.1	0.003
Race/ethnicity						
American Indian	512	58 (9.8%)	501	38 (6.7%)	1.8	0.18
Asian	462	31 (5.9%)	517	33 (6.4%)	0.53	0.82
Black	399	39 (8.1%)	422	23 (4.7%)	2.35	0.13
Hispanic	2,061	146 (6.9%)	2,347	114 (4.5%)	5.43	0.02
Pacific Islander	415	33 (7.1%)	424	26 (5.8%)	0.32	0.57
White	12,724	1,657 (13.3%)	12,242	1,009 (8.5%)	11.35	<0.0001
Substance use						
Smoked in past 30 days	823	233 (30.6%)	620	126 (19.7%)	7.53	0.006
Did not smoke in past 30 days	14,701	1,625 (11%)	14,533	1,029 (7.2%)	10.94	0.001
Drank alcohol within past 30 days	1,759	464 (26.4%)	1,387	268 (19.2%)	11.8	<0.0001
Did not drink within the past 30 days	13,746	1,386 (10.2%)	13,704	876 (6.5%)	10.92	0.0010
Body weight status						
Normal weight	12,655	1,620 (12.9%)	11,255	924 (8.3%)	11.03	0.001
Obese (>95th percentile for weight)	1,068	55 (6.1%)	1123	60 (6.3%)	0.015	0.901
Parental education level						
<High school	822	81 (11.3%)	902	60 (7.5%)	2.98	0.09
High school graduate	2,166	311 (14.8%)	2,128	217 (10.5%)	6.17	0.01
Some college	2,657	370 (14%)	2,524	227 (8.4%)	7.36	0.007
Bachelor degree	5,781	642 (11.1%)	5,453	387 (7%)	8.73	0.003
Graduate degree	2,471	301 (12%)	2,457	154 (6.7%)	9.01	0.003
Local health district						
Bear River	1,745	198 (11.7%)	1,773	134 (7.5%)	3.34	0.07
Central	1,007	163 (15.9%)	964	111 (11.5%)	2.51	0.11
Davis	1,678	200 (13.5%)	590	51 (8.2%)	4.51	0.03
Salt Lake	3,984	393 (10.1%)	4,197	263 (6.6%)	7.35	0.007
Southeast	559	54 (8.3%)	597	57 (9.8%)	0.45	0.49
Southwest	1,065	156 (14%)	1,290	140 (9.7%)	2.95	0.09
Summit	424	50 (11.6%)	477	25 (5.3%)	4.02	0.05
Tooele	855	90 (10.8%)	993	74 (7.8%)	1.78	0.18
Tri-County	432	71 (19.7%)	433	45 (12.3%)	1.85	0.17
Utah County	2,177	274 (11.7%)	2,427	135 (6.7%)	5.02	0.03
Wasatch	321	40 (12.9%)	278	24 (9.5%)	0.58	0.44
Weber-Morgan	1,364	173 (14.5%)	1,206	102 (9.7%)	2.36	0.12

PNA: Prevention Needs Assessment survey; SB 41: Senate Bill 41, Utah 2012 [15].

^a^Data provided in the table have been weighted to account for the probability of selection and the distribution of students by sex, grade, and race/ethnicity using iterative proportional fitting.

**Table 2:** Predictors of adolescent self-reported indoor tanning in the PNA after passage of SB 41.

Variable	Odds ratio	95% confidence interval
Sex		
Male	Referent	Referent
Female	3.72	3.05, 4.55
Grade level		
8th grade	Referent	Referent
10th grade	2.16	1.64, 2.84
12th grade	3.95	3.05, 5.15
Race/ethnicity		
Non-white or Hispanic	0.46	0.34, 0.63
White	Referent	Referent
Parent education level		
Less than college education	Referent	Referent
College graduate	0.78	0.65, 0.94
Alcohol use		
No alcohol within 30 days	Referent	Referent
Drank alcohol within 30 days	2.90	2.23, 3.78
Local health district		
Bear River	1.18	0.87, 1.60
Central	1.76	1.30, 2.39
Davis	1.34	1.01, 1.76
Salt Lake	Referent	Referent
Southeast	1.37	0.98, 1.91
Southwest	1.45	1.03, 2.03
Summit	0.57	0.39, 0.83
Tooele	1.07	0.72, 1.58
Tri-County	1.43	0.79, 2.60
Utah County	1.03	0.74, 1.45
Wasatch	1.24	1.04, 1.49
Weber-Morgan	1.31	1.00, 1.71

PNA: Prevention Needs Assessment survey; SB 41: Utah Senate Bill 41 (2012) [15].
